# Supplementary figures and images for: Dynamic gene expression profiles during postnatal development of porcine subcutaneous adipose
Source: PeerJ. 2016 Mar 10;4:e1768. doi: 10.7717/peerj.1768 (PMC4793310; doi:10.7717/peerj.1768)

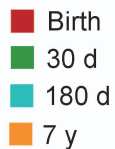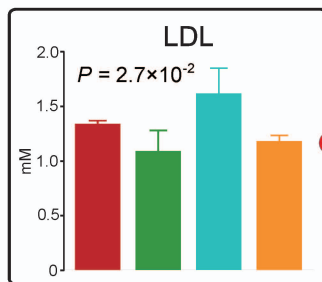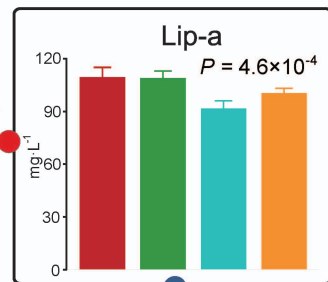

0.685\*

0.629\*

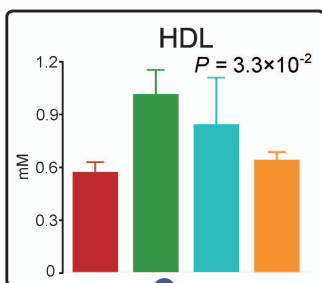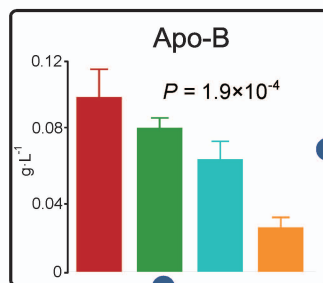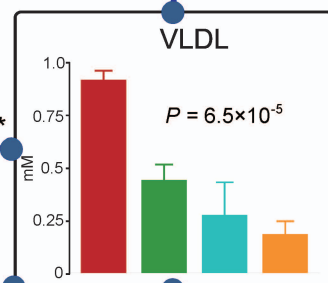

0.774\*\*

0.786\*\*

0.824\*\*\*

0.764\*\*

0.583\*

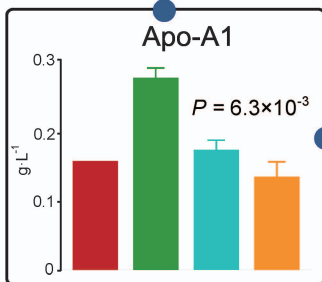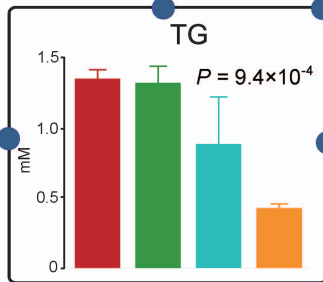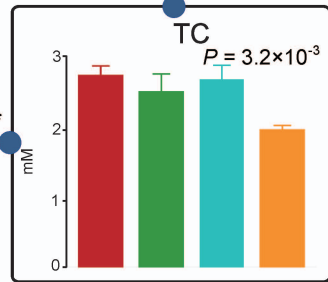

0.601\*

0.809\*\*

Supplement: Supplemental Information 4 — Data normally distributed (Kolmogorov-Smirnov test, P > 0.05). The statistical significance was calculated by one-way repeated-measures ANOVA (n = 3). Values are means ± SD. The Pearson’s correlation was calculated between each pair of indicators. The blue and red connecting lines showed positive and negative correlations (*P < 0.05, **P < 0.01, ***P < 0.001), respectively. [file peerj-04-1768-s004.pdf]

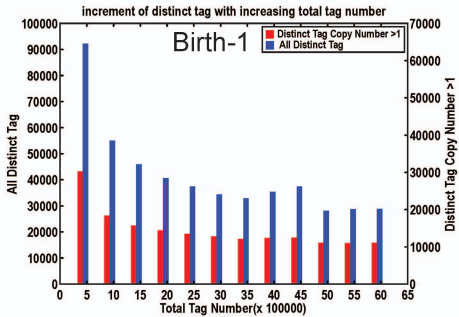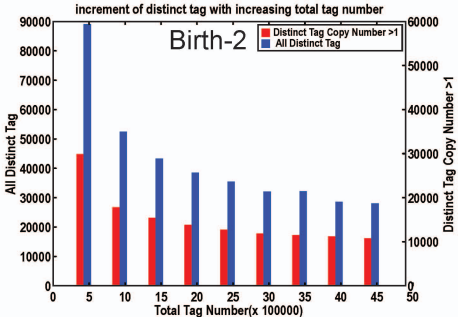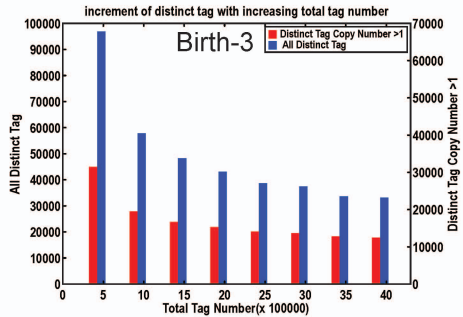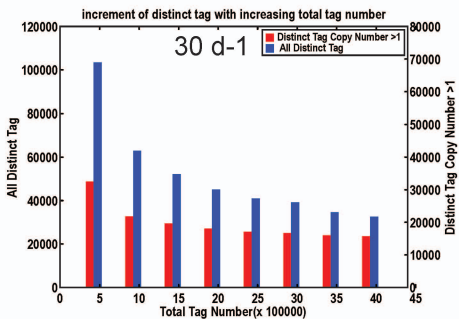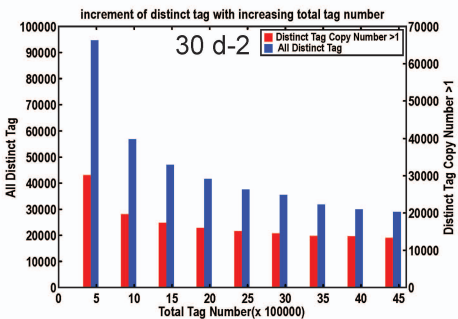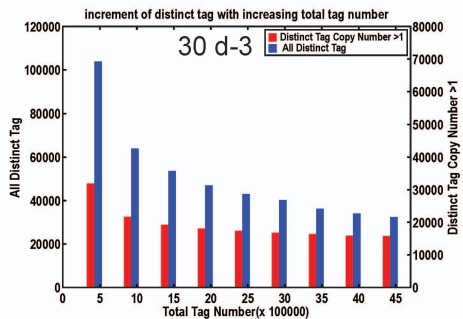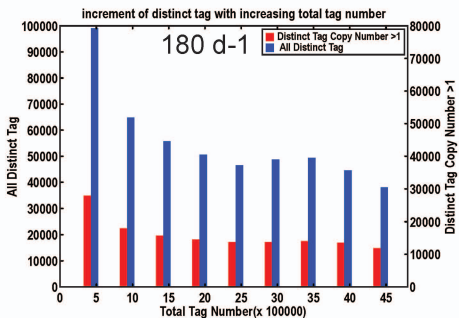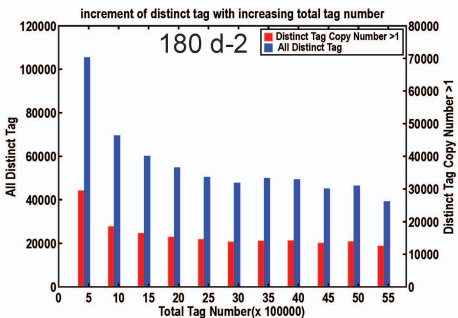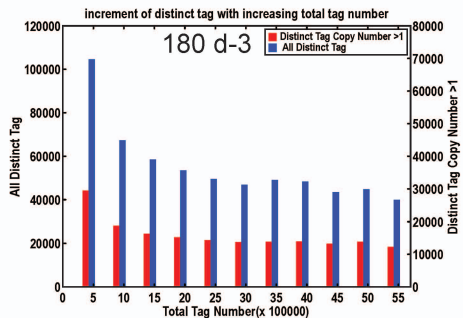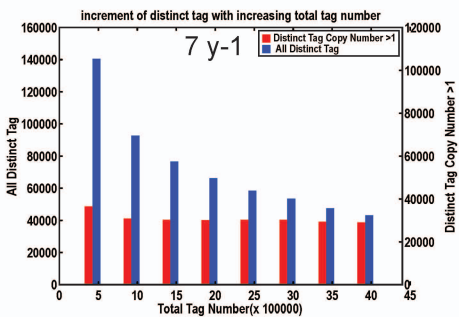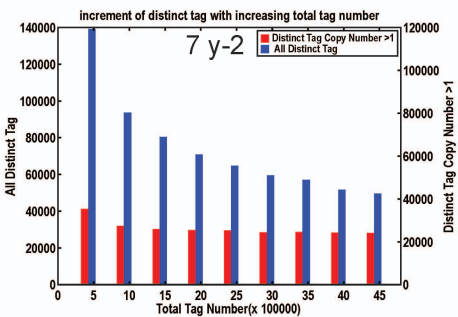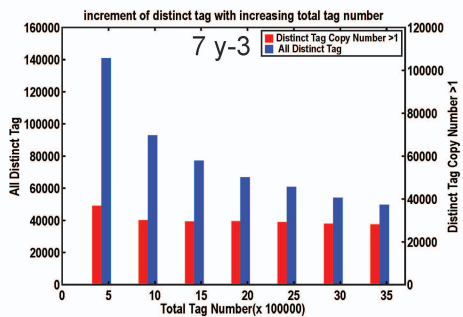

Supplement: Supplemental Information 5 — Saturation analysis of the capacity of libraries demonstrated that newly emerging distinct tags were gradually reduced with increase in total sequence tags when the number of sequencing tags was large enough. When the number of sequencing tags reached one million, library capacity approached saturation. [file peerj-04-1768-s005.pdf]

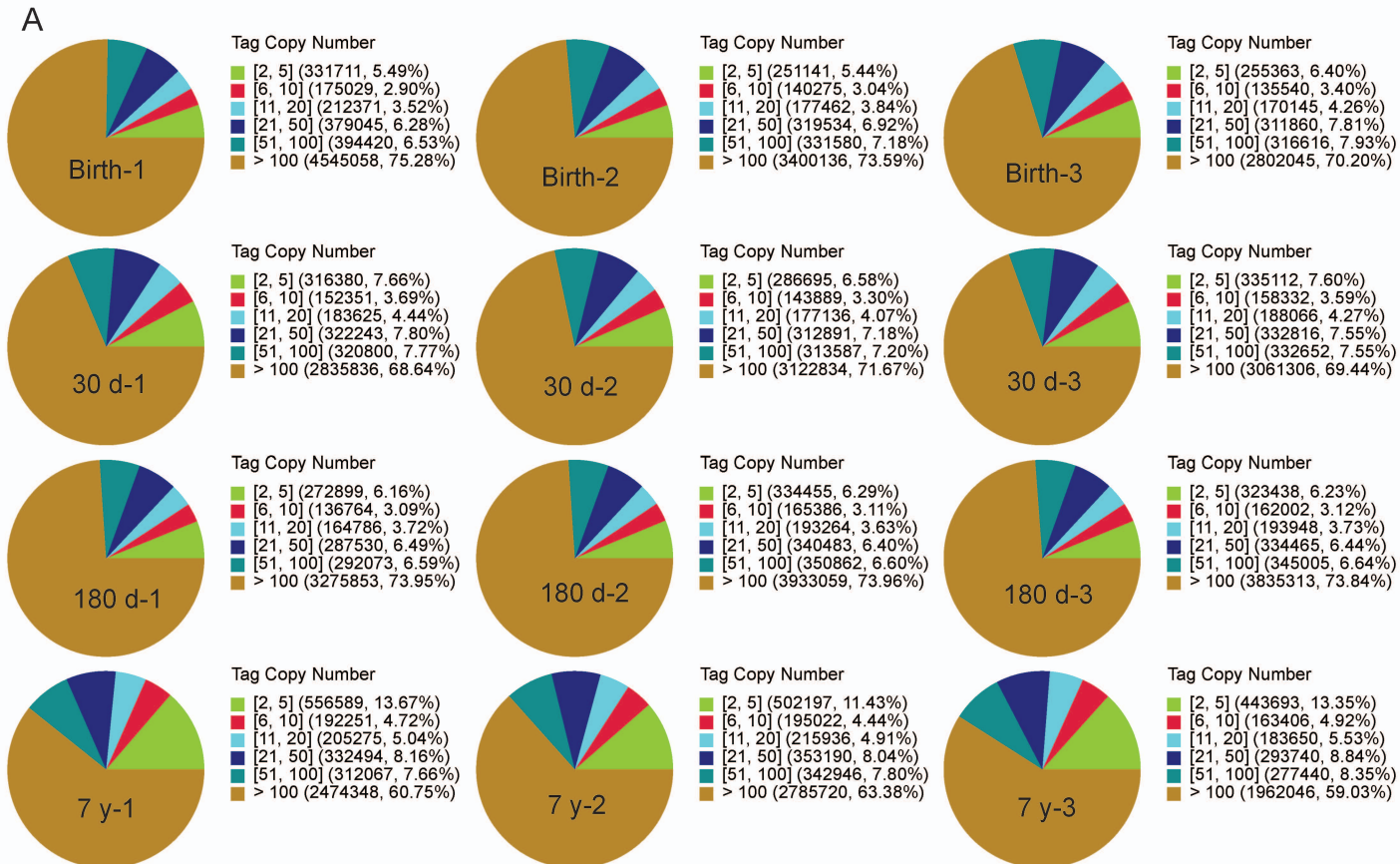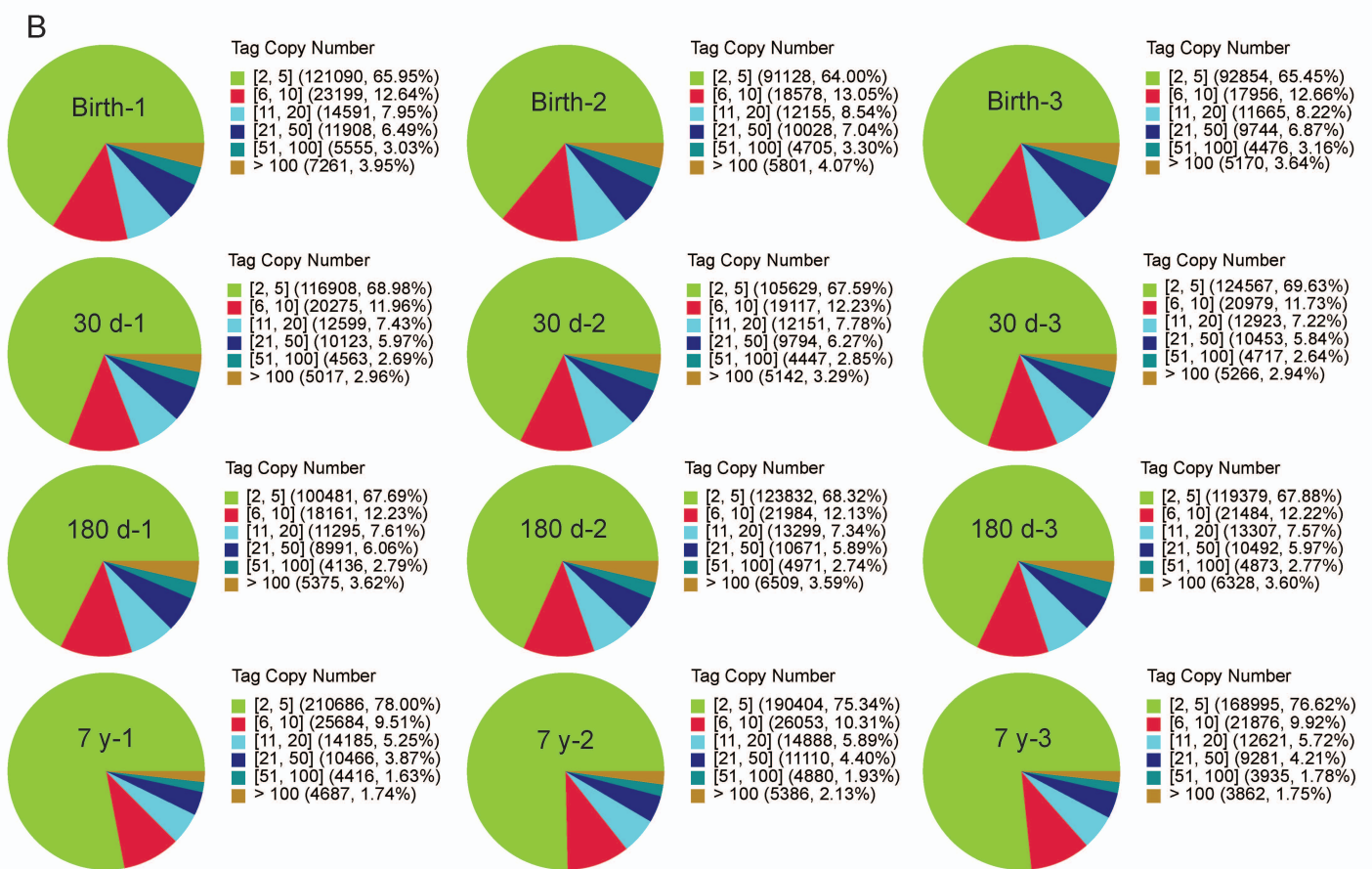

Supplement: Supplemental Information 7 — (A) Distribution of total clean tags. (B) Distribution of distinct clean tags. The distribution of total clean tags and distinct clean tags in each library showed similar patterns; high-expression tags with more than 100 copy numbers comprised 70.1% of the total clean tags, but their distribution did not even reach 2.96% of the distinct clean tags, whereas low-expression tags with copy numbers smaller than 5 accounted for more than 70.5% of the distinct tag distribution. [file peerj-04-1768-s007.pdf]

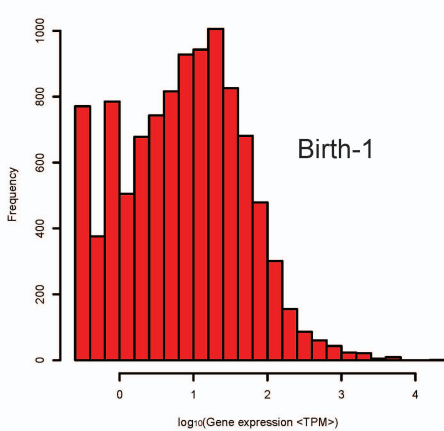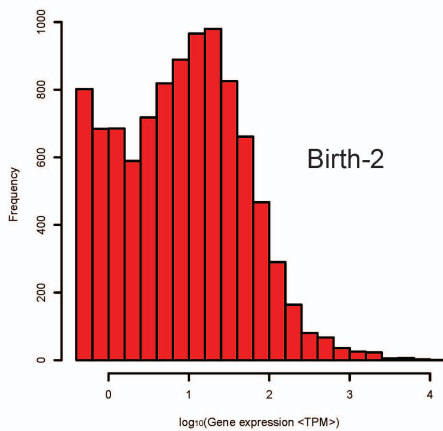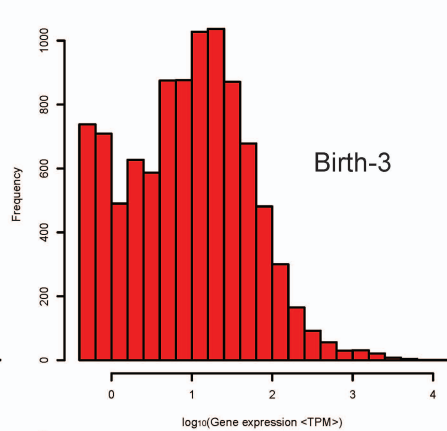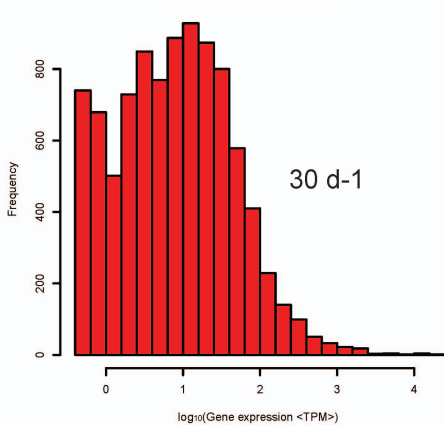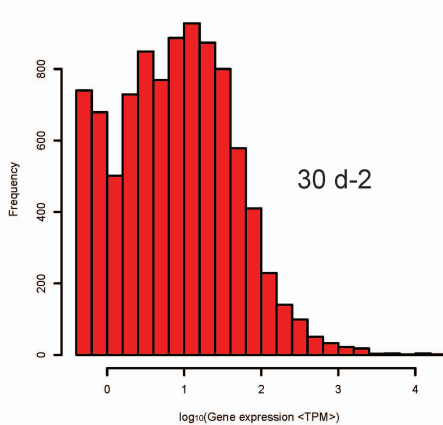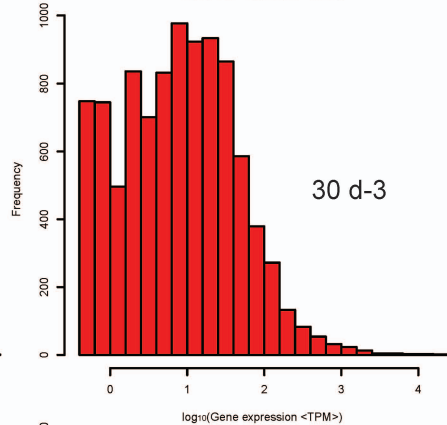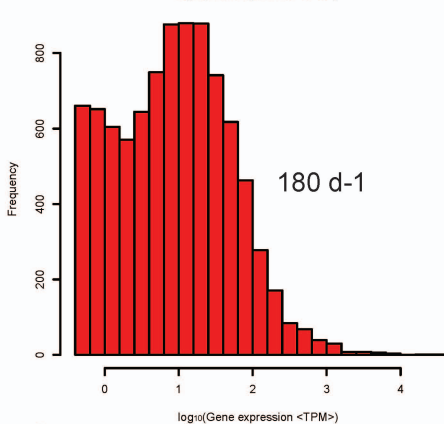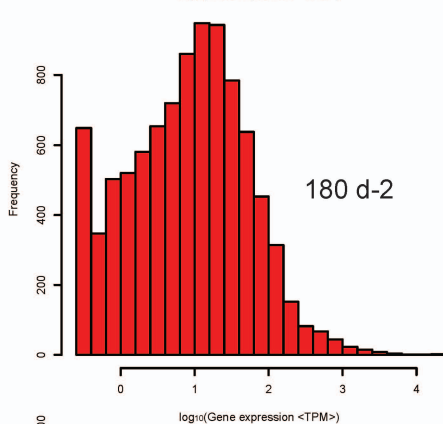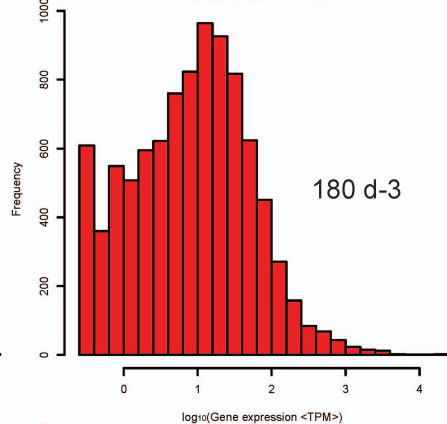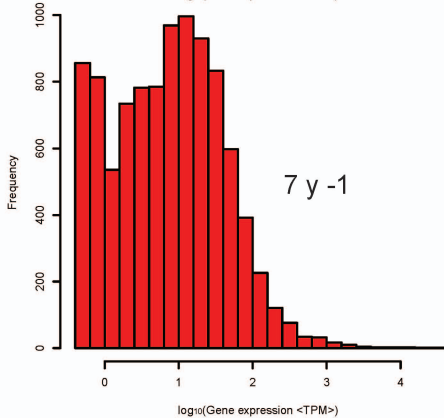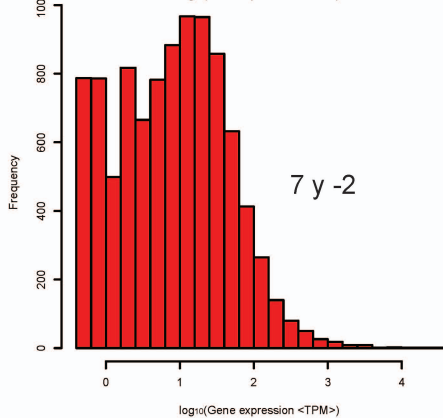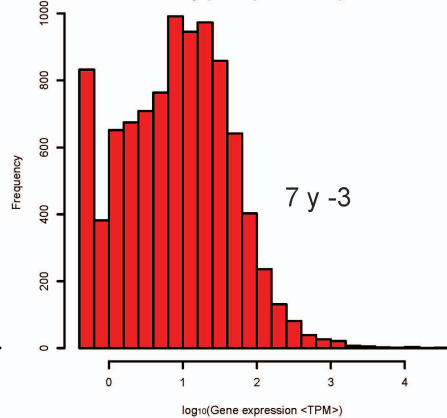

Supplement: Supplemental Information 8 [file peerj-04-1768-s008.pdf]

□ DGE

—○— Q-PCR

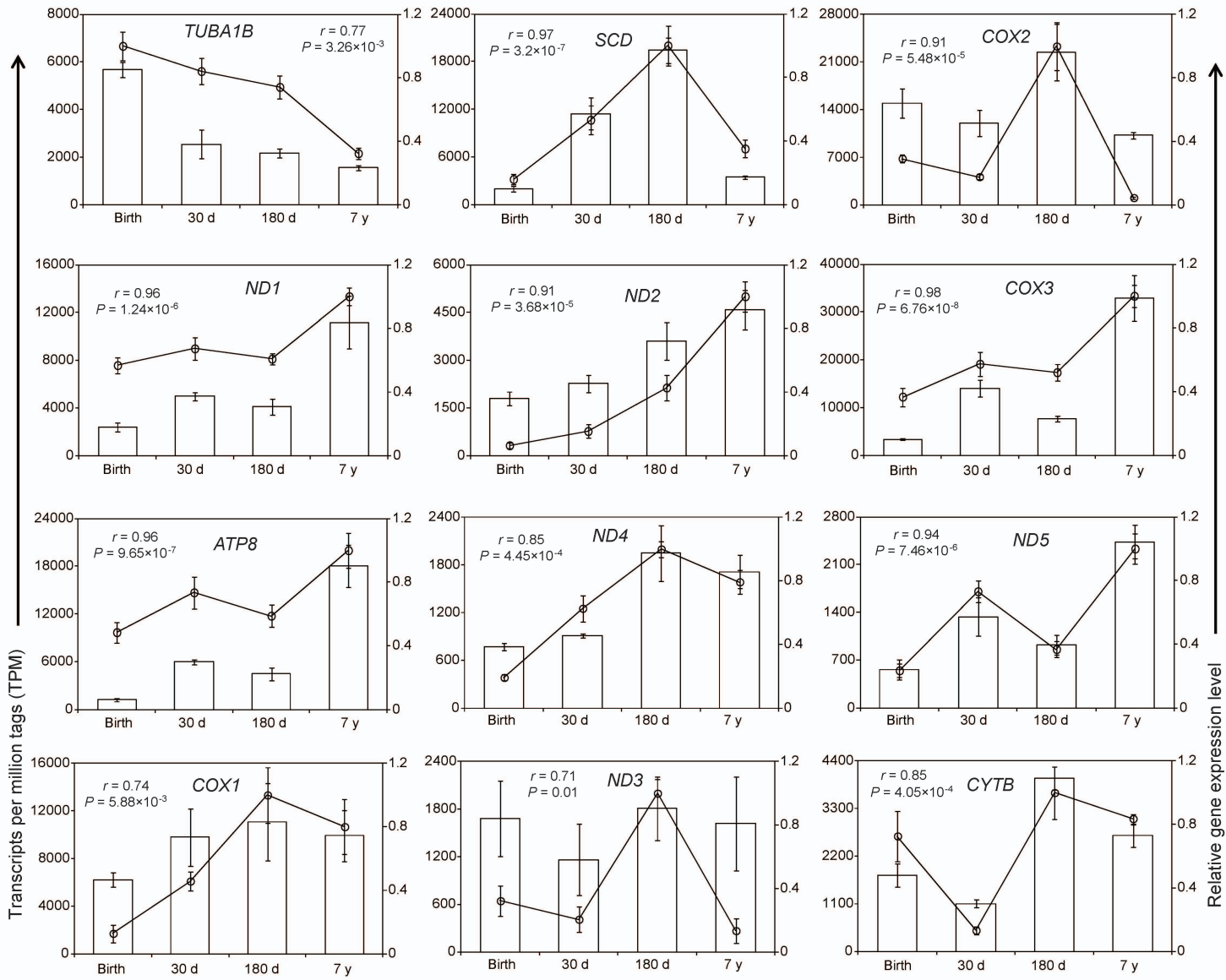

Supplement: Supplemental Information 9 — The data presented in Y-axis indicated the relative mRNA expression of both DGE and q-PCR. Datas are means ± SD. The Pearson correlation coefficient (r) and the corresponding significance value (P) were shown above the columns. [file peerj-04-1768-s009.pdf]

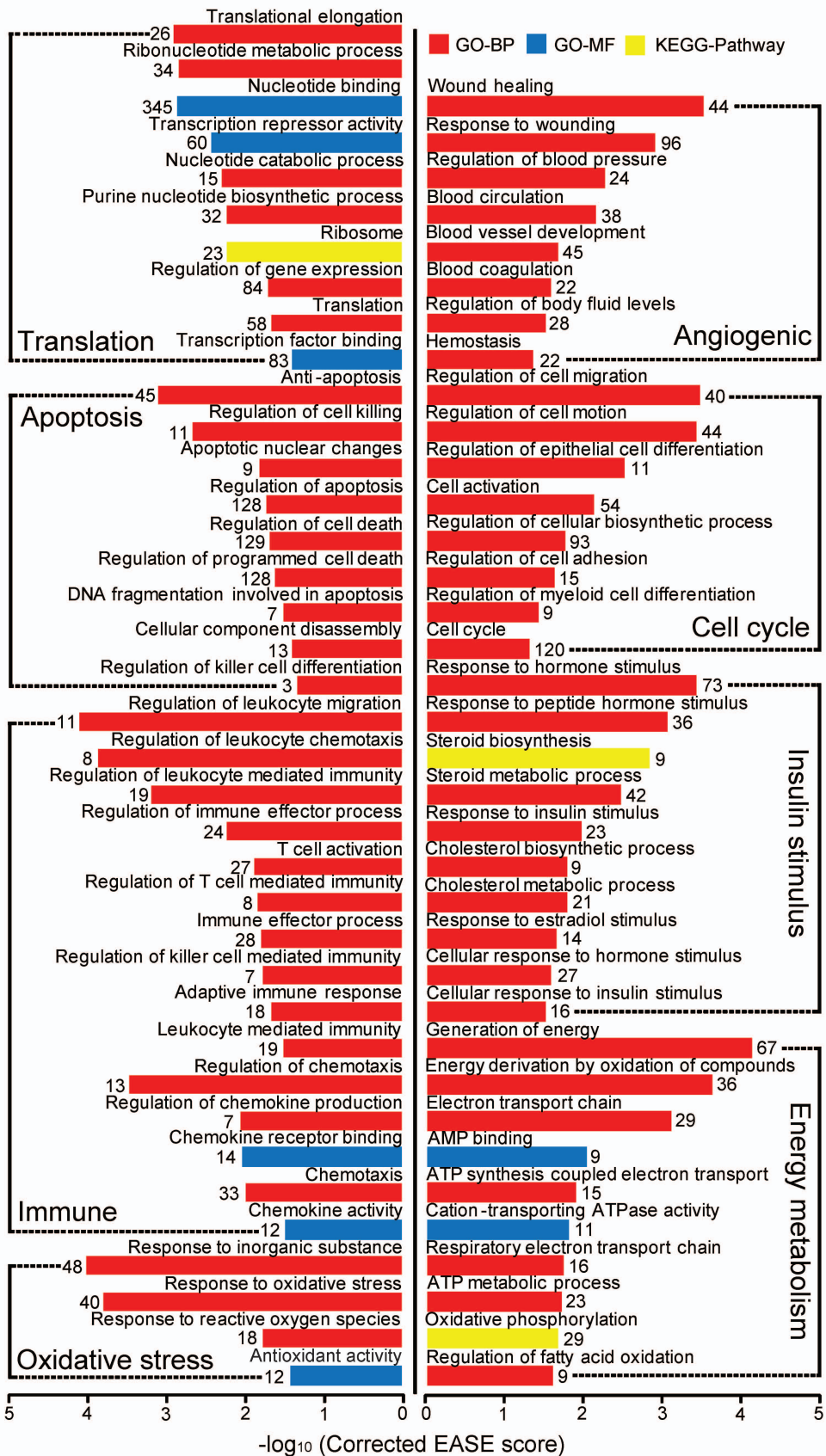

Supplement: Supplemental Information 10 — The EASE score, which indicated the significance of the comparison, was calculated by Benjamini-corrected modified Fisher’s exact test. BP, biological process; MF, molecular function. [file peerj-04-1768-s010.pdf]

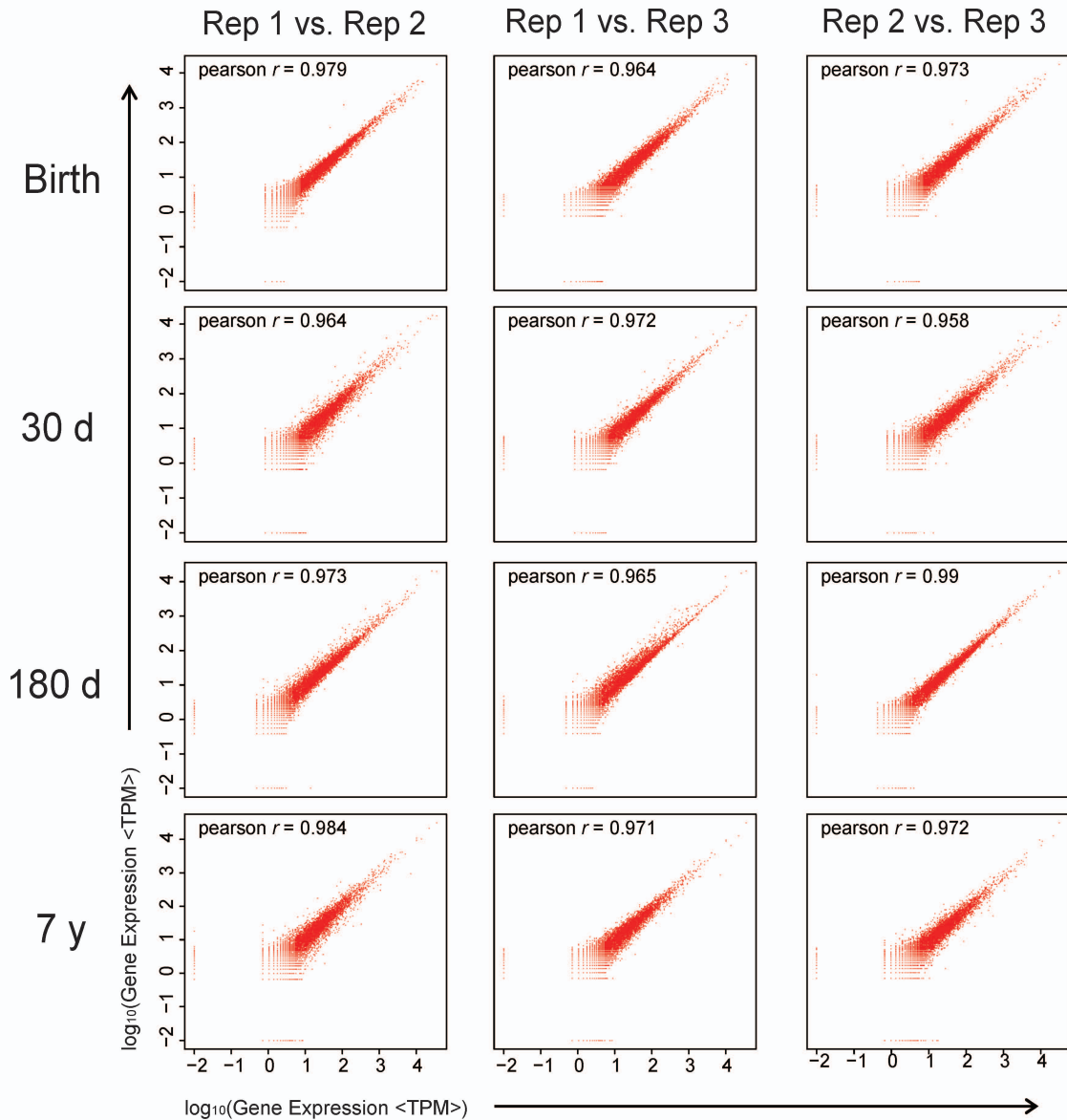

Supplement: Supplemental Information 11 — A scatter plot and Pearson’s correlation revealed a correlation between the log10 of mRNA expression of each biological replicate. [file peerj-04-1768-s011.pdf]

**A**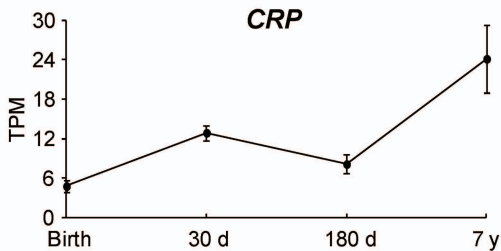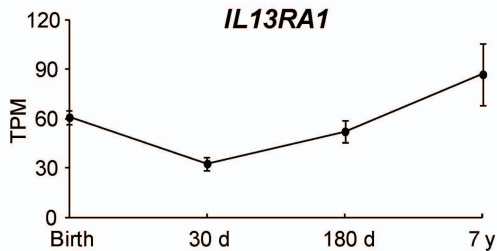**B**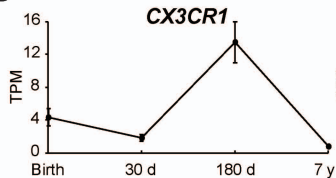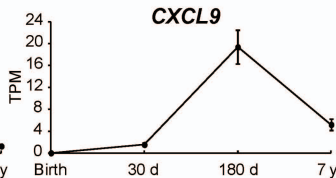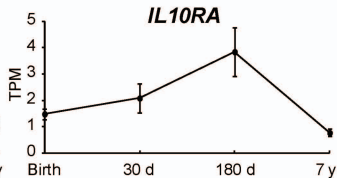**C**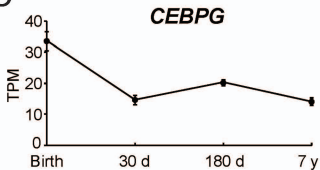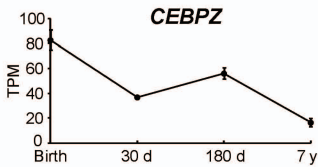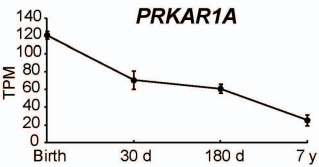**D**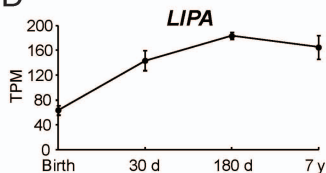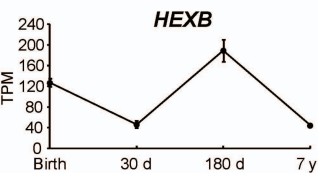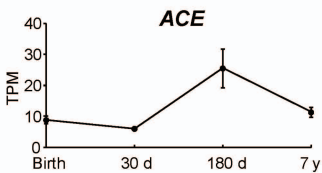

Supplement: Supplemental Information 12 [file peerj-04-1768-s012.pdf]

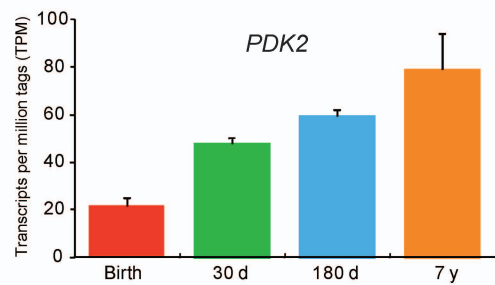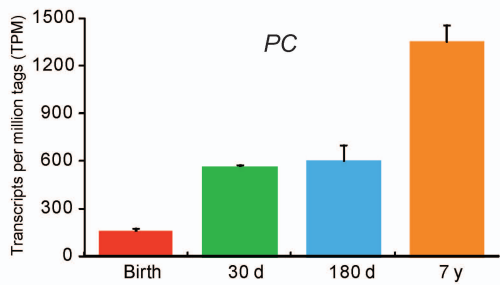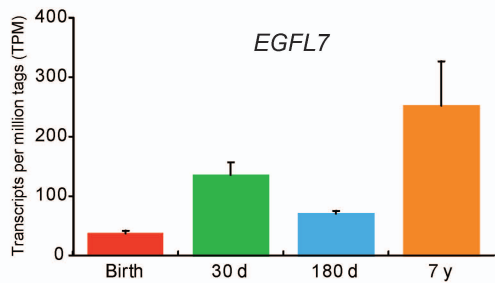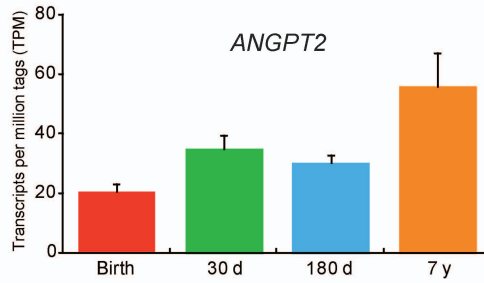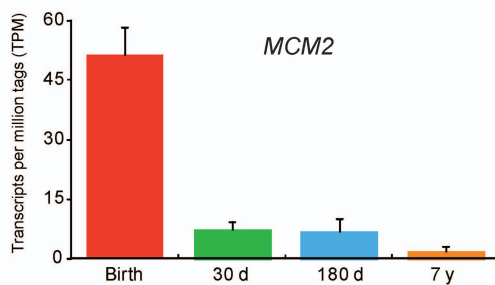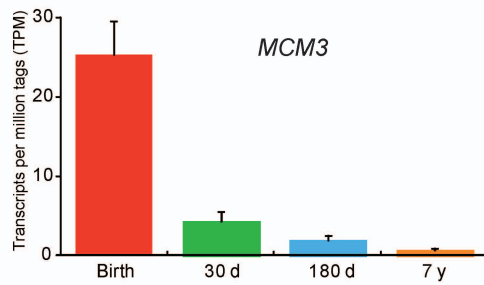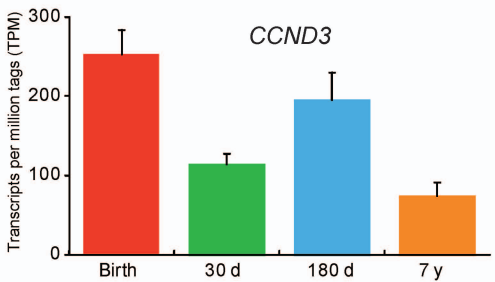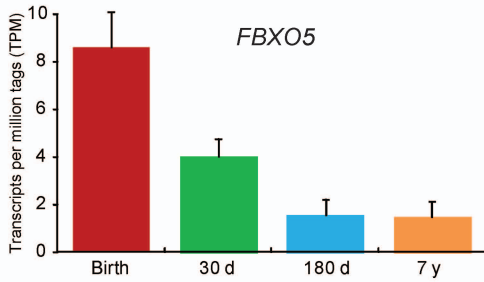

Supplement: Supplemental Information 13 [file peerj-04-1768-s013.pdf]
