# Supplementary material for: Dynamic gene expression profiles during postnatal development of porcine subcutaneous adipose
Source: PeerJ. 2016 Mar 10;4:e1768. doi: 10.7717/peerj.1768 (PMC4793310; doi:10.7717/peerj.1768)
Supplement: Supplemental Information 6 — The rate of increase of all genes identified and genes identified by unambigous tags declined as the size of the library increased. When the library size reached two million, we could identify 45% and 35% of all genes and genes identified by unambigous tags, respectively. At this time, library capacity approached saturation. [file peerj-04-1768-s006.pdf]

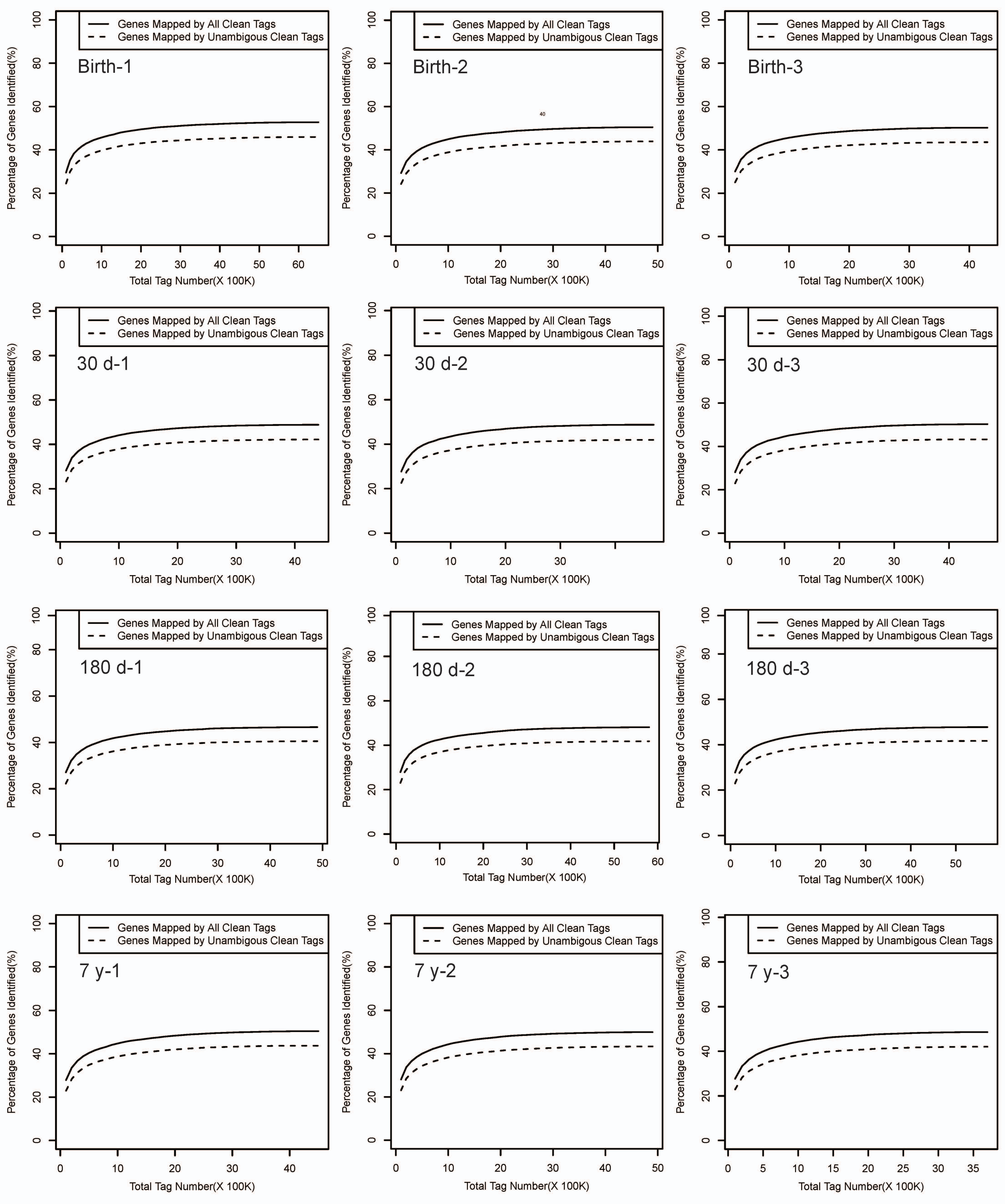

Genes Mapped by All Clean Tags
Genes Mapped by Unambigous Clean Tags
Birth-1
Percentage of Genes Identified(%)
Total Tag Number(X 100K)
Genes Mapped by All Clean Tags
Genes Mapped by Unambigous Clean Tags
Birth-2
Percentage of Genes Identified(%)
Total Tag Number(X 100K)
Genes Mapped by All Clean Tags
Genes Mapped by Unambigous Clean Tags
Birth-3
Percentage of Genes Identified(%)
Total Tag Number(X 100K)
Genes Mapped by All Clean Tags
Genes Mapped by Unambigous Clean Tags
30 d-1
Percentage of Genes Identified(%)
Total Tag Number(X 100K)
Genes Mapped by All Clean Tags
Genes Mapped by Unambigous Clean Tags
30 d-2
Percentage of Genes Identified(%)
Total Tag Number(X 100K)
Genes Mapped by All Clean Tags
Genes Mapped by Unambigous Clean Tags
30 d-3
Percentage of Genes Identified(%)
Total Tag Number(X 100K)
Genes Mapped by All Clean Tags
Genes Mapped by Unambigous Clean Tags
180 d-1
Percentage of Genes Identified(%)
Total Tag Number(X 100K)
Genes Mapped by All Clean Tags
Genes Mapped by Unambigous Clean Tags
180 d-2
Percentage of Genes Identified(%)
Total Tag Number(X 100K)
Genes Mapped by All Clean Tags
Genes Mapped by Unambigous Clean Tags
180 d-3
Percentage of Genes Identified(%)
Total Tag Number(X 100K)
Genes Mapped by All Clean Tags
Genes Mapped by Unambigous Clean Tags
7 y-1
Percentage of Genes Identified(%)
Total Tag Number(X 100K)
Genes Mapped by All Clean Tags
Genes Mapped by Unambigous Clean Tags
7 y-2
Percentage of Genes Identified(%)
Total Tag Number(X 100K)
Genes Mapped by All Clean Tags
Genes Mapped by Unambigous Clean Tags
7 y-3
Percentage of Genes Identified(%)
Total Tag Number(X 100K)
